# Supplementary material for: Hybridization between Alpine Ibex and Domestic Goat in the Alps: A Sporadic and Localized Phenomenon?
Source: Animals (Basel). 2022 Mar 17;12(6):751. doi: 10.3390/ani12060751 (PMC8944563; doi:10.3390/ani12060751)
Supplement: Supplementary file 1 [file animals-12-00751-s001.zip › animals-1577195-supplementary.pdf]

Alpine Ibex - Hybridization network

This form is part of a project focused on Alpine ibex hybridization phenomenon.

Our main scope is to collect all the data available on suspected Alpine ibex hybridizations on the Alps, map their distribution in time and space and organize data in a review paper. A further scope is to raise interest by veterinary authorities and conservation agencies on the tight cohabitation between Alpine ibex and feral goats, an undesirable phenomenon that we perceive on the increase at the Western Alps scale.

If you have directly observed or are aware of observations of suspected / confirmed Alpine ibex hybrids, please fill this form. In case you have observed more than one suspected hybrid, please fill one form for each of the individuals observed.

All sensitive data (name of the person who is reporting the hybrid, email address, etc) will remain completely anonymous and will be stored according to the GDPR 2016/679. We plan to publish the results of this study, but will not include any information that would identify you, although if you wish to be acknowledged, we'll be pleased to mention your contribution in the announced scientific paper. Your participation in this survey is voluntary. You may refuse to take part in the research or exit the survey at any time without penalty.

All data collected will be assessed and processed by our experts. Visual data might be used as material for the paper, while documents on genetics will be kept and stored as confidential. None of the material will be used without the consent of the owner.

This project was conceived by the University of Turin (Department of Veterinary Science) and coordinated in collaboration with Gran Paradiso National Park.

If you need more details on the project, please contact Luca Rossi (luca.rossi@unito.it) or Barbara Moroni (barbara.moroni@unito.it)

1) Email:

2) Name of the person who refers the individual:

3) Country of the hybrid sighting:
- Italy
- France
- Swizterland
- Austria
- Germany
- Slovenia

4) When has the suspected hybrid been observed? Please specify the date or the year of the first sighting?

5) Where has the suspected hybrid been observed? Please specify the place of first sighting at the municipality or administrative division level, if available

6) According to your knowledge, is the suspected hybrid still present in the sighting area?
   - yes
   - no
   - Don't know

7) If you have GPS coordinates of the sighting, please report them here in form of Latitude / Longitude

8) You are reporting:
   - A confirmed case of Alpine ibex hybridization (by genetic analysis)
   - A suspected case of Alpine ibex hybridization based on morphological characteristics

9) What type of evidence do you have?
   - Written evidence (report, published paper, scientific poster, conference abstract)
   - Visual evidence (video, photo, posts on social media etc)
   - Oral evidence (personal communication/anecdotal)

10) If you are reporting a visual evidence that has been taken from a social media/other sources (e.g. photo on Facebook), please specify the source (type of social media, name of the person, date).

11) Are there any documents on the genetic analysis that confirm the hybridization?
   - yes
   - no

12) According to your knowledge, the suspected hybrid is:
   - a solitary individual
   - an individual observed with a group of feral goats
   - an individual observed with a group of domestic goats
   - an individual observed with a group of Alpine ibexes
   - don't know

13) Are or were there any goats grazing near the hybrid localization?
   - yes
   - no
   - don't know

14) Are or were there any abandoned/feral goats near the hybrid localization?
   - yes
   - no
   - don't know

15) Final free comments & notes:

16) Are you going to send any relevant support documentation (doc, pdf, image, etc)? In case you wish to do so, please send it to the the email address: barbara.moroni@unito.it
    - yes
    - no

17) If you have read the informed consent form (see initial description of this form), understand the information contained in this informed consent form, and agree to participate in this study, including the use of attached supporting documentation for the elaboration and publication of a scientific paper. click "yes". If you do not wish to participate, click "no".
    - yes
    - no

18) I wish to be aknowledged in the scientific paper:

- Yes
- no
